# Transcriptional Responses of a Bicarbonate-Tolerant Monocot, *Puccinellia tenuiflora,* and a Related Bicarbonate-Sensitive Species, *Poa annua,* to NaHCO_3_ Stress

**DOI:** 10.3390/ijms16010496

**Published:** 2014-12-29

**Authors:** Shio Kobayashi, Hina Satone, Engkong Tan, Hiroyuki Kurokochi, Shuichi Asakawa, Shenkui Liu, Tetsuo Takano

**Affiliations:** 1 Asian Natural Environmental Science Center (ANESC), the University of Tokyo, Nishitokyo-shi, Tokyo 188-0002, Japan; E-Mails: kobayashi@anesc.u-tokyo.ac.jp (S.K.); kurokochi@anesc.u-tokyo.ac.jp (H.K.); 2 Graduate School of Agricultural and Life Sciences, the University of Tokyo, Bunkyo-ku, Tokyo 113-8657, Japan; E-Mails: asatone@mail.ecc.u-tokyo.ac.jp (H.S.); tanengkong@gmail.com (E.T.); asakawa@mail.ecc.u-tokyo.ac.jp (S.A.); 3 Alkali Soil Natural Environmental Science Center (ASNESC), Northeast Forestry University, Harbin Hexing Road, Harbin 150040, China; E-Mail: shenkuiliu@nefu.edu.cn

**Keywords:** *Puccinellia tenuiflora*, *Poa annua*, RNA-seq, alkaline salt stress, bicarbonate

## Abstract

*Puccinellia tenuiflora* is an alkaline salt-tolerant monocot found in saline-alkali soil in China. To identify the genes which are determining the higher tolerance of *P. tenuiflora* compared to bicarbonate sensitive species, we examined the responses of *P. tenuiflora* and a related bicarbonate-sensitive *Poeae* plant, *Poa annua*, to two days of 20 mM NaHCO_3_ stress by RNA-seq analysis. We obtained 28 and 38 million reads for *P. tenuiflora* and *P. annua*, respectively. For each species, the reads of both unstressed and stressed samples were combined for *de novo* assembly of contigs. We obtained 77,329 contigs for *P. tenuiflora* and 115,335 contigs for *P.* *annua*. NaHCO_3_ stress resulted in greater than two-fold absolute expression value changes in 157 of the *P. tenuiflora* contigs and 1090 of *P. annua* contigs. Homologs of the genes involved in Fe acquisition, which are important for the survival of plants under alkaline stress, were up-regulated in *P. tenuiflora* and down-regulated in *P. annua*. The smaller number of the genes differentially regulated in *P. tenuiflora* suggests that the genes regulating bicarbonate tolerance are constitutively expressed in *P. tenuiflora*.

## 1. Introduction

Soil salinity and sodicity are major environmental stresses faced by crops. In Songnen Plain in Northeast China, about 3.73 × 10^6^ ha contain elevated levels of alkaline salt, and the area is expanding at a rate of 1.4% annually [1,2]. In alkaline soils, plant stress factors include excess Na^+^ and HCO_3_^−^/CO_3_^2^^−^ ions and high pH. In highly alkaline areas, only a small number of plants can grow.

Recently some transcriptome analyses under bicarbonate stress have been performed on both bicarbonate-tolerant and bicarbonate-sensitive species. The bicarbonate tolerant species include *Leymus chinensis* [3], *Puccinellia tenuiflora* [4,5,6,7], *Tamarix hispida* [8,9], *Limonium bicolor* [10] and the sensitive species include soybean [11], maize [2], *Lotus japonicus* [12] and flax [13]. However, most of these studies examined only a single species, which makes it difficult to compare the responses of bicarbonate-tolerant and bicarbonate-sensitive species.

*Puccinellia tenuiflora* (Griseb.) Scrib. et Merr. is a graminaceous plant found in saline-alkali soil in Songnen Plain, China. It is tolerant to both neutral and alkaline salts, and its tolerance mechanisms have been extensively studied. *P. tenuiflora* is able to maintain a high K^+^/Na^+^ ratio due to a high K^+^/Na^+^ selectivity of its plasma membrane [14] and a high ability to limit Na^+^ influx in the roots [15]. In addition, its leaves exude salts with wax through the stomata [16]. *P. tenuiflora* is able to maintain high photosynthetic activity under low NaCl stress, possibly through the activity of antioxidant enzymes [17]. *P. tenuiflora* also accumulates and exudes citric acid under alkaline salt stress, and secretes it from the roots, where it may adjust the pH of the rhizosphere [18]. Several *P. tenuiflora* genes that are presumably involved in the response to alkaline salt stress have been cloned and characterized [19,20,21,22,23,24,25,26]. EST [4,6,7], microarray [5,6] and proteomics [27] analyses have identified genes in various categories (metabolism, transcription regulation, signal transduction, transport *etc.*) that are presumably involved in the responses of *P. tenuiflora* to salt stress. However, it is unclear whether these responses are specific to *P. tenuiflora* or are shared with sensitive species.

Here, to obtain insights into the molecular mechanisms of alkaline salt tolerance, we compared the transcriptomes of *Puccinellia tenuiflora* and the salt-sensitive *Poa annua* L. (both of the tribe *Poeae*).

## 2. Results

### 2.1. Bicarbonate Stress Tolerance Test of P. annua

**Figure 1:**
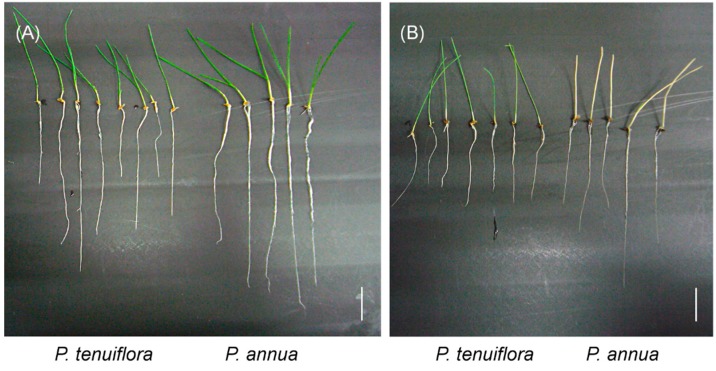
Stress tolerance of *P. tenuiflora* (left) and *P. annua* (right). Seedlings of *P. annua* and *P. annua* were hydroponically grown for nine days and were transferred to the nutrient solution containing 0 (**A**); or 300 mM NaHCO_3_ (**B**), and were grown for another five days. White bars = 1 cm

*P. annua* grew faster than *P. tenuiflora* under the control condition. Under the stress of 300 mM NaHCO_3_, *P. tenuiflora* survived but *P. annua* did not (Figure 1B), indicating that *P. annua* was a suitable species for a comparison of bicarbonate stress tolerance.

**Figure 2:**
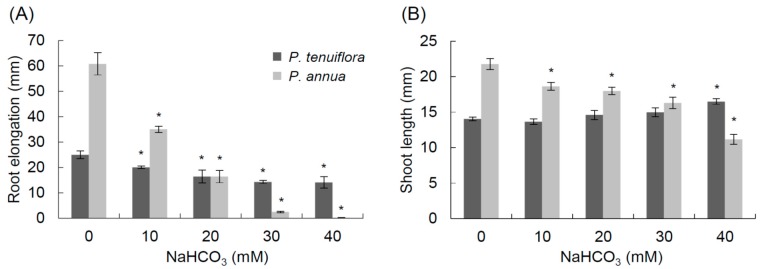
Root elongation during NaHCO_3_ treatment (**A**) and shoot length after five days of treatment (**B**) of *P. tenuiflora* and *P. annua* under the indicated concentrations of NaHCO_3_. Values show the average of three plates, each containing 7–10 seedlings. The error bars represent standard error (SE) and * indicate values that are significantly different from those under 0 mM NaHCO_3_ (*p* < 0.05 in Student’s *t* test).

*P. annua* roots continued to elongate at NaHCO_3_ concentrations up to 30 mM, but stopped growing and the root tips turned black at 40 mM NaHCO_3_ (Figure 2A). Root growth of *P. tenuiflora* was reduced at NaHCO_3_ concentrations as low as 10 mM, but was not stopped by concentrations up to 40 mM NaHCO_3_. Based on these results, we used 20 mM NaHCO_3_ for the RNA-seq analysis.

### 2.2. De Novo Assembly of P. tenuiflora and P. annua Transcripts

After two sequencing runs using the same cDNA libraries (see Experimental Section 4.3 for details), we obtained 29,619,901 reads for *P. tenuiflora* and 47,700,089 reads for *P. annua*. After *de novo* assembly, 77,329 and 115,335 contigs were obtained for *P. tenuiflora* and *P. annua*, respectively. Setting stringent parameters for assembly to avoid *trans* chimeras resulted in relatively short contigs. Species distributions of the top BLASTX hits for the contigs of each species are shown in Figure S1. For both species, the grass *Aegilops tauscii* was the species with the most hits and the species distributions were very similar.

### 2.3. Read Mapping and Gene Annotation

The reads from the second run were mapped to the assembled contigs to calculate the expression values, and the expression values were compared between the control and the stressed samples. The numbers of the contigs whose expressions changed >2- or <0.5-fold under NaHCO_3_ stress compared to the control were 1090 in *P. annua* and 157 in *P. tenuiflora* (Table S2). In this table, fold changes of ∞ and −∞ indicate transcripts that were expressed only under the NaHCO_3_ condition or only under the control condition, respectively. However, transcripts detected only under one condition or the other may include artifacts caused by misassembled contigs.

**Figure 3:**
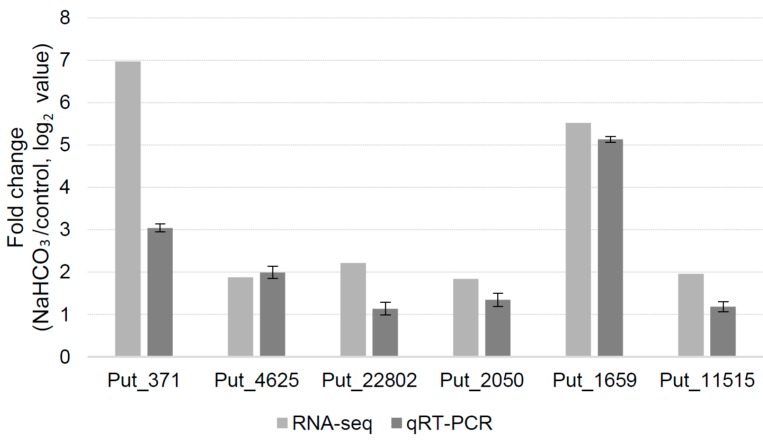
Fold change in expression values (expressed as Log_2_ values) of selected genes as determined by RNA-seq analysis and real-time quantitative RT-PCR. RNAs extracted from the same plant samples were used for the two analyses and *tubulin* was used as an internal control for the RT-PCR.

The expressions of some of the genes which were differentially regulated in the RNA-seq analysis were checked by qRT-PCR. For each of the genes, the qRT-PCR results confirmed the RNA-seq results (Figure 3).

## 3. Discussion

### 3.1. Effects of NaHCO_3_ Stress on P. tenuiflora and P. annua

*P. annua* was sensitive to NaHCO_3_ (Figure 1 and Figure 2). NaHCO_3_ concentrations as low as 40 mM killed the root tip of *P. annua* and totally blocked its elongation (Figure 2). On the other hand, roots of *P. tenuiflora* continued to elongate under 40 mM NaHCO_3_ treatment. The plants could survive 300 mM NaHCO_3_ stress treatment for five days, suggesting that the ability to protect root tips from bicarbonate stress is important for survival.

Twenty mM NaHCO_3_ treatment on agar plates had less effect on shoot growth than on root growth in both species. This finding is contrary to the finding that carbonate stress increased the root/shoot ratio of pea [28]. However, it should be noted that the stress treatment in this study was performed in sealed plates. The air inside the plates would have had a high CO_2_ concentration generated from bicarbonate, which might have affected the respiration and photosynthesis of the shoots.

### 3.2. RNA-Seq Analysis and de Novo Assembly

*De novo* assembly of the reads obtained in the first RNA-seq revealed the presence of many *trans* chimeras [29] that were later incorrectly assigned as up- or down-regulated genes, probably as the result of high expressions of rRNAs. Although rRNA removal tools and PCR duplicate removal tools have not been commonly used in other transcriptome studies, in the present study, they seemed to reduce the number of *trans* chimeras in the up- or down-regulated contigs. Setting stringent parameters for assembly also helped to reduce the number of chimeras, although it also resulted in the frequent appearance of possibly the same gene products in the up- and down-regulated genes lists. Because our goal was to identify the kinds of genes that are differentially regulated by bicarbonate stress, we concluded that multiple counts of the same gene products would have less effect on our results than the occurrence of *trans* chimeras. It is also possible that the contigs that yielded the same BLAST hits are actually orthologs or splice variants.

#### 3.2.1. Genes Differentially Regulated in *P. annua* under NaHCO_3_ Treatment

Significantly more genes were differentially regulated in *P. annua* than in *P. tenuiflora* (Table S2), suggesting that *P. annua* was more severely stressed by NaHCO_3_ than *P. tenuiflora*. Some of the up-regulated genes were probable homologs of genes involved in stress tolerance and defense mechanisms such as *glutathione S-transferase*, disease resistance protein *RPM1* and mitochondrial chaperone *BCS1-B* as well as genes involved in metabolism such as *aspartic proteinase nepenthesin-2* and *patatin* group A-3 (Table 1 and “poaup_hit” tab in Table S2). Homologs of genes encoding *flavonoid 3'-monooxygenase-like protein* and *anthocyanidin 5,3-O-glucosyltransferase-like protein* were up-regulated, suggesting that secondary metabolites have a role in protecting against bicarbonate stress.

**Table 1:** Genes that were up-regulated under NaHCO_3_ treatment (fold change > 2, FDR-corrected *p*-value < 0.05). The annotations were assigned by BDA (Blast2GO Descriptor Annotator), showing the words most frequently counted of all blast hit descriptions (https://www.blast2go.com/b2gsupport/faqs).

Contig	Annotation	Fold Change (EDGE Test)
***P. annua***		
**Poa_27190**	Glutathione *S*-transferase	11.34
**Poa_3657**	High affinity nitrate transporter	53.21
**Poa_12387**	Anthocyanidin 5,3- *O*-glucosyltransferase-like	5.09
**Poa_688**	ABC transporter b family member 4-like	62.27
**Poa_2744**	Aspartic proteinase nepenthesin-2	7.68
**Poa_18988**	Cytochrome p450	54.50
**Poa_15731**	Disease resistance protein RPM1	18.52
**Poa_4898**	Flavonoid 3-monooxygenase-like	14.47
**Poa_6299**	Mitochondrial chaperone BCS1-B	2.93
**Poa_8595**	Patatin group A-3	3.97
**Poa_2480**	Phosphate transporter	176.58
**Poa_8940**	Phosphoenolpyruvate carboxykinase	54.00
**Poa_12214**	Potassium channel SKOR	6.51
**Poa_25791**	Probable WRKY transcription factor 70-like	16.62
**Poa_19228**	Subtilisin-like protease	9.35
***P. tenuiflora***		
**Put_2050**	Boron transporter	3.58
**Put_11515**	Carbonic anhydrase	3.90
**Put_1357**	High-affinity nitrate transporter-like	3.53
**Put_763**	Leucine-rich repeat receptor-like protein kinase at2g19210-like	3.48
**Put_2064**	Long chain acyl-CoA synthetase 4-like	5.03
**Put_4625**	Metal-nicotianamine transporter YSL6	3.68
**Put_22802**	Nicotianamine aminotransferase A-like	4.65
**Put_1383**	Phosphate transporter	25.87
**Put_1973**	Phosphoenolpyruvate carboxykinase	7.47
**Put_7021**	Sucrase-like protein	6.08
**Put_3399**	Sulfate transporter	3.21
**Put_4027**	Thionin-like peptide	6.82
**Put_10418**	Zinc transporter	5.74

EDGE = Empirical analysis of digital gene expression [30].

High affinity nitrate transporters and predicted inorganic phosphate transporters were up-regulated in both *P. annua* and *P. tenuiflora* under bicarbonate stress. There have been conflicting reports on the effect of HCO_3_^−^ on the uptake of NO_3_^−^ [31,32,33,34,35,36,37] and on phosphorus status in plants [38,39], and the roles of these transporters under bicarbonate stress are not clear. However, the genes for nitrate transporters in alkaline salt-tolerant ecotype of *Lotus japonicus* has been reported to be up-regulated under bicarbonate stress [40]. Phosphoenolpyruvate carboxykinase (*PEPCK*) was strongly induced by NaHCO_3_ in both species, in agreement with previous studies [11,41], and may have a role in maintaining homeostasis of intracellular CO_2_/H_2_CO_3_/HCO_3_^−^ and/or organic acids. However, its function in the response to NaHCO_3_ stress is unclear.

Transcripts involved in metal acquisition and homeostasis were down-regulated only in *P. annua* (Table S2). Down-regulated transcripts included homologs of iron-phytosiderophore transporter, *nicotianamine aminotransferase A* (*NAAT-A*) and *nicotianamine synthase* (*NAS*). Bicarbonate stress induces Fe chlorosis in plants [42,43]. High pH and HCO_3_^−^ ion have been suggested to impede the solubility of Fe in the rhizosphere and plant apoplast, making Fe unavailable for plant cells [42,43,44]. NASs and NAATs are involved in synthesis of phytosiderophores, which are members of the mugineic acid family. Overexpression of *OsNAS2* led to a higher tolerance of Fe deficiency and to high pH [45], and gene expression of *NAAT* was reported to be induced by Fe deficiency in barley [46]. Metal-nicotianamine transporter YSL (yellow stripe 1-like) transports Fe-chelated phytosiderophore complexes from rhizosphere into root cells. Putative homologs of transporters such as high-affinity potassium transporters [47] and zinc-induced facilitator-like 1 [48] were also down-regulated in *P. annua*, suggesting that NaHCO_3_ disrupts the ion homeostasis in *P. annua*. Down-regulation of *β-expansin 1a precursor* and *cellulose synthase* only in *P. annua* may partly account for the reduced root growth of the species under NaHCO_3_ stress.

#### 3.2.2. Genes Differentially Regulated in *P. tenuiflora* under NaHCO_3_ Treatment

Metal-nicotianamine transporter and *NAAT-A*, which were down-regulated in *P. annua*, were up-regulated in *P. tenuiflora*. The difference in the responses of genes related to Fe-acquisition in *P. tenuiflora* and *P. annua* (Table 1 and Table S2) suggests that *P. tenuiflora* is better able to cope with Fe deficiency under alkaline salt stress. A putative homolog of *BOR-like 2* was also induced in *P. tenuiflora*. BOR is involved in the export of boron from the cytoplasm to the apoplast [49]. Although 10 mM NaHCO_3_ treatment did not reduce the concentration of boron in leaves of *Lotus tenuis* [40], boron transporters may have an important function in saline-alkali fields since boron is said to be adsorbed to the soil in the presence of CaCO_3_ under high pH [50]. Other genes that were up-regulated only in *P. tenuiflora* included putative homologs of genes involved in metabolism (e.g., sucrase-like protein) and defense (e.g., thionin-like peptide [51]). Metabolic responses and defense responses must be important in both *P. annua* and *P. tenuiflora* under NaHCO_3_ stress, but the genes involved seem to be different.

Transcripts down-regulated in *P. tenuiflora* include members of families that are known to be involved in other stress responses (e.g., cytochrome p450 and WRKY transcription factors; Table 2 and Table S2). *PutHKT2;1*, which is described as a “salt transporter” in Table 2 and which has a high-affinity Na^+^-K^+^ symport activity [21], was also down-regulated. Thus, down-regulation of *PutHKT2;1* might contribute to maintaining intracellular ion homeostasis under weak bicarbonate stress conditions.

**Table 2:** Genes that were down-regulated under NaHCO_3_ treatment (fold change < −2, FDR-corrected *p*-value < 0.05).

Contig	Annotation	Fold Change (EDGE Test)
***P. annua***		
**Poa_40361**	2'-Deoxymugineic-acid 2'-dioxygenase-like	−3.90
**Poa_7047**	Actin	−3.31
**Poa_13081**	Ammonium transporter	−2.94
**Poa_24077**	β-Expansin 1a precursor	−8.33
**Poa_8466**	Cellulose synthase	−3.48
**Poa_9099**	GDSL esterase lipase At5g45910-like	−2.24
**Poa_2507**	High-affinity potassium transporter	−2.80
**Poa_21856**	Iron-phytosiderophore transporter	−3.51
**Poa_36815**	Mate efflux family protein chloroplastic-like	−6.77
**Poa_2955**	Nicotianamine aminotransferase A-like	−4.99
**Poa_64935**	Nicotianamine synthase 3	−90.37
**Poa_58054**	Protein zinc induced facilitator-like 1-like	−50.8
**Poa_41522**	STOP1	−9.44
**Poa_6256**	Urea active transporter 1	−2.47
**Poa_9416**	Wall-associated receptor kinase 2-like	−3.47
***P. tenuiflora***		
**Put_32521**	Cytochrome p450 716b1-like	−3.72
**Put_28631**	Sodium transporter	−8.04
**Put_52287**	WRKY transcription factor	−5.15

## 4. Experimental Section

### 4.1. Plant Materials, Growth Conditions and Stress Treatments

Seeds of *P. tenuiflora* were collected in an alkaline soil area located in North-East China. Seeds of *Poa annua* were kindly provided by Masaru Ogasawara at University of Utsunomiya, Japan. Seeds were surface-sterilized by washing with 70% *v*/*v* ethanol for 5 min. and subsequently with 50% *v*/*v* NaClO for 15 min. Growth chamber was maintained at 28 °C during the day and 22 °C at night while the daily photoperiod of 350–400 µmol·m^−2^·s^−1^ was 12 h.

To compare the survival rates under strong alkaline salt stress, the surface-sterilized seeds were sown in tap water and were grown for nine days. The seedlings were transferred to nutrient solution containing 6 mg/L (NH_4_)_2_SO_4_, 2 mg/L K_2_SO_4_, 8.2 mg/L MgSO_4_, 2.3 mg/L KNO_3_, 7.5 mg/L Ca(NO_3_)_2_, 3.1 mg/L KH_2_PO_4_, 10 mg/L Fe-EDTA with 0 or 300 mM NaHCO_3_. Photos were taken after 5 days. The experiment was repeated twice and similar results were obtained.

For measuring the shoot and root length under weak alkaline salt stress, the surface-sterilized seeds were sown on 0.8% *w*/*v* agar plates containing the nutrient solution described above. After 4 days (*P. annua*) and 9 days (*P. tenuiflora*) of germination, the plants were transferred to 0.8% *w*/*v* agar plates containing the nutrients mentioned above and 0, 10, 20, 30 or 40 mM NaHCO_3_. The *P. tenuiflora* seedlings were allowed to germinate longer because they grow more slowly than *P. annua* seedlings. Shoot length and root elongation were measured after 5 days.

For RNA-seq analysis, surface-sterilized seeds were hydroponically grown for 19 days. Water was changed every 3–4 days. The plants were transferred to the nutrient solution containing 0 or 20 mM NaHCO_3_ and harvested after 48 h. Three biological replicates with >100 plants each were obtained.

### 4.2. RNA Extraction and cDNA Library Construction

Plants harvested in the previous subsection were separated into shoots and roots, frozen immediately using liquid nitrogen and ground to fine powder. In the first sequencing, mRNA was extracted directly from powdered tissue samples with a Dynabeads^®^ DIRECT™ Micro Kit (Life Technologies, Carlsbad, CA, USA) following the manufacturer’s instructions. In the second sequencing, total RNA was extracted from frozen root samples using an RNeasy Plant Mini Kit (QIAGEN, Venlo, The Netherlands). mRNA was then isolated from total RNA with the Dynabeads Kit (See subsection 4.3 for details; Life Technologies). mRNA samples were fragmented, reverse transcribed and amplified to make barcoded whole transcriptome libraries using Ion Total RNA-seq Kit v2 (Life Technologies). Yield and size distribution of the fragmented RNA and the amplified cDNA were checked using an Agilent 2200 Tapestation with High Sensitivity RNA ScreenTape^®^ and High Sensitivity D1000 ScreenTape^®^ (Agilent Technologies, Palo Alto, CA, USA) at each step. For libraries whose peak sizes of amplified cDNAs were <200 bp (all the libraries in the first run and *P. annua* control #2, *P. tenuiflora* control #3 and *P. tenuiflora* control #1 in the second run), cDNAs with the sizes around 280 bp were selected using E-Gel^®^ SizeSelect™ Agarose Gel (Life Technologies). Ion OneTouch™ System with Ion PI™ Template OT2 200 Kit v3 (Life Technologies) was used to prepare enriched, template-positive Ion PI™ Ion Sphere Particles. The whole library preparation and sequencing were performed twice from the same sample sets, obtaining 24 libraries from 12 sample sets (see the next section for detail).

### 4.3. Next Generation Sequencing and Data Analysis

Sequencing was performed using the Ion Proton™ System with an Ion PI™ Sequencing 200 Kit v3 (Life Technologies) following the manufacturer’s instructions. The sequencing results are summarized in Table S1 . The numbers of raw reads were different in the different libraries, which was caused by an error in diluting the cDNA libraries during the library preparation step. Since the expression values calculated in the RNA-seq analysis described later were normalized to the number of total reads in the given libraries, the difference in the number of total reads should not greatly impact the conclusions. Sequencing results were imported into CLC Genomic Workbench 7.5 (CLC bio, Aarhus, Denmark) as FASTQ files for further analysis. On CLC Genomic Workbench, the raw reads with the quality score less than 0.05 were trimmed using the “Trim Sequences” tool. Reads shorter than 15 bp were discarded. The average quality scores of the trimmed reads were 22.89 for *P. annua* and 23.08 for* P. tenuiflora*. In the first trial of RNA-seq, the trimmed reads were *de novo* assembled just after this step. However, BLASTN analysis revealed that many of the resulting contigs were *trans* chimeras [29] resulting from misassembly of different gene products into one contig. Many of them seemed to be chimeras of rRNA and other transcripts, so in the second trial we tried to reduce rRNA contamination by extracting mRNAs from total RNA instead of directly from tissue samples. This way rRNA contamination was reduced substantially (Table S1), but not totally removed. Thus we used SortMeRNA (ver. 1.99 beta, [52]) to filter out probable rRNA contamination from the trimmed reads. The average quality scores of the trimmed reads from the second run were 23.60 for *P. annua* and 23.27 *P. tenuiflora*. The trimmed reads were exported as FASTQ files from CLC Genomics Workbench. rRNA was removed from the reads with SortMeRNA with the databases supplied with the software (silva-euk-18s-database-id95.fasta & silva-euk-28s-database-id95.fasta). The “length of the sliding window” option was set (“-L 14”) to allow reads longer than 14 bp. Default settings were used for other parameters. Reads not assigned as rRNA were imported back to CLC Genomics Workbench. The same process was applied to the reads from the first run, and the reads from the two runs were combined to be used for *de novo* assembly on CLC Genomics Workbench. The reads were *de novo* assembled for each plant species using 12 libraries each with the word and bubble size automatically set by the software to yield contigs (word size 23 and 24 bp, bubble size 108 and 119 bp for *P. annua* and *P. tenuiflora*, respectively). Minimum contig length was set to 200 bp. To raise the precision of the contigs, the trimmed reads were mapped back to the resulting contigs with the following settings: mismatch cost 2, insertion cost 3, deletion cost 3, length fraction 0.8 and similarity fraction 0.95. The contig sequences were updated occasionally according to the mapped reads. After the removal of probable PCR duplicates from the trimmed reads using Duplicate Read Removal plugin (ver. 1.0, beta, CLC bio) of the CLC Genomics Workbench, expression analysis was performed with RNA-Seq Analysis Tool of CLC Genomic Workbench for each sample groups of the second run, using *de novo* assembled contigs as references. Parameters for read mapping were set as follows: Mismatch cost 2, insertion cost 3, deletion cost 3, length fraction 0.8, similarity fraction 0.8 and maximum number of hits for a read 10. Expression levels were compared between control groups and stressed groups using Empirical Analysis of DGE [30,53] tool on CLC Genomics Workbench 7.5. Reads per kilobase of exon per million mapped reads (RPKM) was also calculated. Contigs whose absolute fold changes were >2 (FDR-corrected *p*-values <0.05) by EDGE test were selected as up-regulated or down-regulated transcripts. For those contigs, homology searches using BLASTX (BLAST+ ver. 2.2.30, NCBI) against the *Poaceae* (taxid: 4479) protein sequences from NCBI nr protein database and annotation with Blast2GO (ver. 2.7.2; [54]) were performed. Summary of rRNA removal and duplicate removal are shown in Table S1.

The FASTQ files of the raw reads and the sequences of differentially regulated contigs were deposited to DDBJ Sequence Read Archive (BioProject ID: PRJDB3227).

### 4.4. Real-Time PCR Analysis

Selected contigs that were differentially regulated by the NaHCO_3_ stress were examined by real-time PCR analysis. Total RNA was extracted from the same plant root samples that were used in RNA-seq analysis with RNeasy Plant Mini Kit (QIAGEN), and reverse transcribed using High-Capacity RNA-to-cDNA™ Kit (Life Technologies) following the manufacturer’s instructions. The cDNA was diluted 20 times and 1 μL of the diluted cDNA was used as the template for quantitative RT-PCR analysis. SYBR^®^ *Premix Ex Taq*™ II (Tli RNase H Plus) (TaKaRa, Shiga, Japan) and StepOne™ Real-Time PCR System (Applied Biosystems, Framingham, MA, USA) were used for the PCR. A *tubulin* gene from *P. tenuiflora* was used as an internal standard to normalize the expression data [21]. The primer pairs (Table S3) were designed to yield products with the sizes of 80–200 bp based on the contig sequences. The PCR was performed as follows: 95 °C for 30 s, followed by 40 cycles of 95 °C for 5 s and 60 °C for 30 s. The experiments were carried out in triplicate. Ten-fold serial dilution of 0.2× cDNA mixture of all the samples were used for drawing the standard curve.

## 5. Conclusions

The present RNA-seq analysis revealed the transcriptome of *P. tenuiflora* on a larger scale than did previous studies, and showed significant differences in the numbers of genes that respond to NaHCO_3_ stress between related NaHCO_3_-tolerant and NaHCO_3_-sensitive species. The tolerant *P. tenuiflora* seemed to adapt to bicarbonate stress by regulating a small number of genes including those important for Fe acquisition, which suggests that *P. tenuiflora* has a high tolerance to NaHCO_3_, even when grown under unstressed conditions.

## Supplementary Materials

Supplementary materials can be found at http://www.mdpi.com/1422-0067/16/01/0496/s1.
